# Supplementary figures and images for: Comparative study on chloroplast genomes of three Hansenia forbesii varieties (Apiaceae)
Source: PLoS One. 2023 Jun 1;18(6):e0286587. doi: 10.1371/journal.pone.0286587 (PMC10234559; doi:10.1371/journal.pone.0286587)

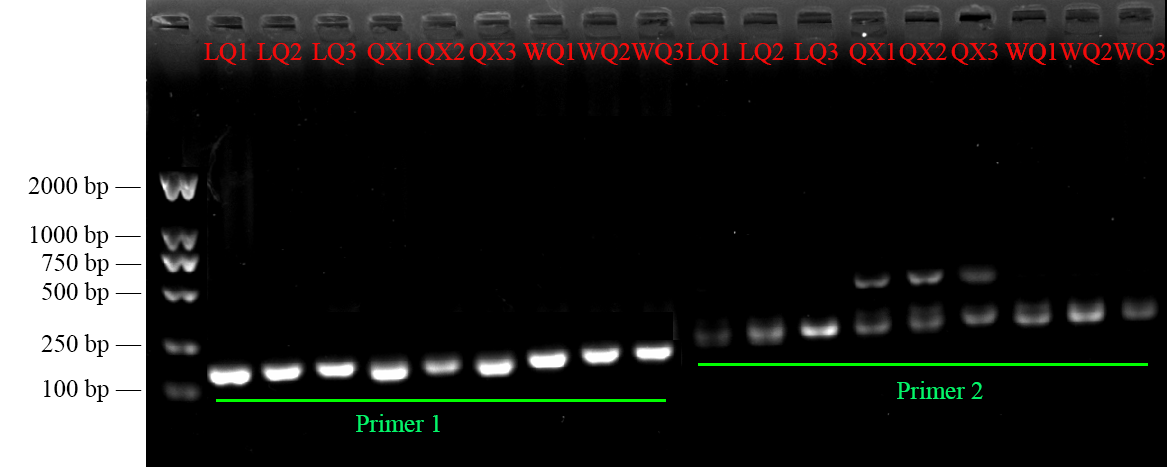

Supplement: S1 Fig — Lane M was the marker of DL 2000. The lanes from left to right corresponded to products of LQ1, LQ2, LQ3, QX1, QX2, QX3, WQ1, WQ2, and WQ3, respectively. (TIF) [file pone.0286587.s005.tif]
