# Supplementary material for: Comparative study on chloroplast genomes of three Hansenia forbesii varieties (Apiaceae)
Source: PLoS One. 2023 Jun 1;18(6):e0286587. doi: 10.1371/journal.pone.0286587 (PMC10234559; doi:10.1371/journal.pone.0286587)
Supplement: Attachment — Submitted filename: PONE-D-23-04874.docx [file pone.0286587.s006.docx]

PLOS ONE

**Comparative study on chloroplast genomes of three Hansenia forbesii varieties (Apiaceae)**

--Manuscript Draft--

| **Manuscript Number:** | PONE-D-23-04874 |
| --- | --- |
| **Article Type:** | Research Article |
| **Full Title:** | Comparative study on chloroplast genomes of three Hansenia forbesii varieties (Apiaceae) |
| **Short Title:** | Comparative study on chloroplast genomes of three Hansenia forbesii varieties |
| **Corresponding Author:** | sun zhirong  Beijing University of Chinese Medicine Beijing, Beijing CHINA |
| **Keywords:** | Hansenia forbesii H.Boissieu, Variety differentiation, Chloroplast genome, Phylogenetic tree, Apiaceae |
| **Abstract:** | To find the gene hypervariable regions of three varieties of Hansenia forbesii H. Boissieu and determine their phylogenetic relationship, the chloroplast (cp) genomes of these three varieties were firstly sequencing by the Illumina hiseq platform. In this study, we assembled the complete cp genome sequences of Hansenia forbesii LQ (156,954 bp), H. forbesii QX (157,181 bp), H. forbesii WQ (156,975 bp). They all contained 84 protein-coding genes, 37 tRNAs, and 8 rRNAs. The hypervariable regions between the three cp genomes were atpF-atpH, petD, and rps15-ycf1.  Phylogenetic analysis showed that H. forbesii LQ and H. forbesii WQ were closely related, followed by H. forbesii QX. This study showed that the three varieties of H. forbesii could be identified by the complete cp genome and provided a new idea for germplasm identification of similar cultivated varieties. |
| **Order of Authors:** | Chenghao Zhu |
|  | Yuan Jiang |
|  | Yu Bai |
|  | Shengjian Dong |
|  | Zhirong Sun |
| **Additional Information:** |  |
| **Question** | **Response** |
| **Financial Disclosure**  Enter a financial disclosure statement that describes the sources of funding for the work included in this submission. Review the [submission guidelines](http://journals.plos.org/plosone/s/submission-guidelines#loc-financial-disclosure-statement) for detailed requirements. View published research articles from [*PLOS ONE*](http://journals.plos.org/plosone/) for specific examples.  This statement is required for submission and **will appear in the published article** if the submission is accepted. Please make sure it is accurate. | the Ministry of Finance and Ministry of Agriculture and Rural Areas: special subsidy of national modern agricultural industrial technology system (cars-21); Gansu Dingxi Science and Technology Project (dx2018n04). |

| **Unfunded studies**  Enter: *The author(s) received no specific funding for this work.*  **Funded studies**  Enter a statement with the following details:   - Initials of the authors who received each award - Grant numbers awarded to each author - The full name of each funder - URL of each funder website - Did the sponsors or funders play any role in the study design, data collection and analysis, decision to publish, or preparation of the manuscript? - **NO** - Include this sentence at the end of your statement: *The funders had no role in study design, data collection and analysis, decision to publish, or preparation of the manuscript.* - **YES** - Specify the role(s) played. |  |
| --- | --- |
| * typeset |  |
| **Competing Interests**  Use the instructions below to enter a competing interest statement for this submission. On behalf of all authors, disclose any [competing interests](http://journals.plos.org/plosone/s/competing-interests) that could be perceived to bias this work—acknowledging all financial support and any other relevant financial or non- financial competing interests.  This statement is required for submission and **will appear in the published article** if the submission is accepted. Please make sure it is accurate and that any funding sources listed in your Funding Information later in the submission form are also declared in your Financial Disclosure statement.  View published research articles from  [*PLOS ONE*](http://journals.plos.org/plosone/) for specific examples. | We declare that there is no conflict of interest regarding the publication of this article. |

| **NO authors have competing interests**  Enter: *The authors have declared that no competing interests exist.*  **Authors with competing interests**  Enter competing interest details beginning with this statement:  *I have read the journal's policy and the authors of this manuscript have the following competing interests: [insert competing interests here]* |  |
| --- | --- |
| * typeset |  |
| **Ethics Statement**  Enter an ethics statement for this submission. This statement is required if the study involved:   - Human participants - Human specimens or tissue - Vertebrate animals or cephalopods - Vertebrate embryos or tissues - Field research   Write "N/A" if the submission does not require an ethics statement.  General guidance is provided below. Consult the [submission guidelines](http://journals.plos.org/plosone/s/submission-guidelines#loc-guidelines-for-specific-study-types) for detailed instructions. **Make sure that all information entered here is included in the Methods section of the manuscript.** | N/A |

| **Format for specific study types**  **Human Subject Research (involving human participants and/or tissue)**   - Give the name of the institutional review board or ethics committee that approved the study - Include the approval number and/or a statement indicating approval of this research - Indicate the form of consent obtained (written/oral) or the reason that consent was not obtained (e.g. the data were analyzed anonymously)   **Animal Research (involving vertebrate animals, embryos or tissues)**   - Provide the name of the Institutional Animal Care and Use Committee (IACUC) or other relevant ethics board that reviewed the study protocol, and indicate whether they approved this research or granted a formal waiver of ethical approval - Include an approval number if one was obtained - If the study involved *non-human primates*, add *additional details* about animal welfare and steps taken to ameliorate suffering - If anesthesia, euthanasia, or any kind of animal sacrifice is part of the study, include briefly which substances and/or methods were applied   **Field Research**  Include the following details if this study involves the collection of plant, animal, or other materials from a natural setting:   - Field permit number - Name of the institution or relevant body that granted permission |  |
| --- | --- |
|  |  |
| **Data Availability**  Authors are required to make all data underlying the findings described fully available, without restriction, and from the time of publication. PLOS allows rare exceptions to address legal and ethical concerns. See the [PLOS Data Policy](http://journals.plos.org/plosone/s/data-availability) and [FAQ](http://journals.plos.org/plosone/s/data-availability#loc-faqs-for-data-policy) for detailed information. | Yes - all data are fully available without restriction |

| A Data Availability Statement describing where the data can be found is required at submission. Your answers to this question constitute the Data Availability Statement and **will be published in the article**, if accepted. |  |
| --- | --- |
| **Important:** Stating ‘data available on request from the author’ is not sufficient. If your data are only available upon request, select ‘No’ for the first question and explain your exceptional situation in the text box. |  |
| Do the authors confirm that all data underlying the findings described in their manuscript are fully available without restriction? |  |
| **Describe where the data may be found in full sentences. If you are copying our sample text, replace any instances of XXX with the appropriate details.**   - If the data are **held or will be held in a public repository**, include URLs, accession numbers or DOIs. If this information will only be available after acceptance, indicate this by ticking the box below. For example: *All XXX files are available from the XXX database (accession number(s) XXX, XXX.*). - If the data are all contained **within the manuscript and/or Supporting Information files**, enter the following: *All relevant data are within the manuscript and its Supporting Information files.* - If neither of these applies but you are able to provide **details of access elsewhere**, with or without limitations, please do so. For example:   *Data cannot be shared publicly because of [XXX]. Data are available from the XXX Institutional Data Access / Ethics Committee (contact via XXX) for researchers who meet the criteria for access to confidential data.*  *The data underlying the results presented in the study are available from (include the name of the third party* | The genome sequence data that support the findings of this study are openly available in GenBank of NCBI at [[https://www.ncbi.nlm.nih.gov]](http://www.ncbi.nlm.nih.gov/) under accession no. ON 208134, ON208135, ON208136. |

| *and contact information or URL).*   - This text is appropriate if the data are owned by a third party and authors do not have permission to share the data.   * typeset |  |
| --- | --- |
| Additional data availability information: |  |

Manuscript	[Click here to access/download;Manuscript;chloroplast genomes of three varieties.doc](https://www.editorialmanager.com/pone/download.aspx?id=32685519&guid=16ee874b-5db8-475f-ba39-37ac828faad0&scheme=1)

1	**Comparative study on chloroplast genomes of three *Hansenia forbesii* varieties (Apiaceae)**

2

## 3 Chenghao Zhu^1^, Yuan Jiang^1^, Yu Bai^1^, Shengjian Dong^2^, Zhirong Sun^1*^

4

5 **1** School of Chinese Materia Medica, Beijing University of Chinese Medicine. Beijing 102488,
6 Beijing, China, **2** College of Applied Technology, Gansu Agricultural University. Lanzhou
7 730500, Gansu, China

8

9	^*^[szrbucm67@163.com](mailto:szrbucm67@163.com)

10

## 11 Abstract

12 To find the gene hypervariable regions of three varieties of *Hansenia forbesii* H. Boissieu and
13 determine their phylogenetic relationship, the chloroplast (cp) genomes of these three varieties
14 were firstly sequencing by the Illumina hiseq platform. In this study, we assembled the complete
15 cp genome sequences of *Hansenia forbesii* LQ (156,954 bp), *H. forbesii* QX (157,181 bp), *H.*
16 *forbesii* WQ (156,975 bp). They all contained 84 protein-coding genes, 37 tRNAs, and 8 rRNAs.
17 The hypervariable regions between the three cp genomes were *atpF*-*atpH*, *petD*, and *rps15*-*ycf1*.
18 Phylogenetic analysis showed that *H. forbesii* LQ and *H. forbesii* WQ were closely related,
19 followed by *H. forbesii* QX. This study showed that the three varieties of *H. forbesii* could be
20 identified by the complete cp genome and provided a new idea for germplasm identification of
21 similar cultivated varieties.

22

23

# 24 1. Introduction

25 *Hansenia forbesii* H.Boissieu is a rare and endangered perennial herb belonging to the genus
26 *Hansenia* (Apiaceae), and the Chinese name is “Kuan-ye-qiang-huo”. The main chemical
27 components isolated from *H. forbesii* are volatile oils, terpenoids, coumarins, sugars and
28 glycosides, alkynes, phenolic acids, steroids, flavonoids, and alkaloids. It has anti-inflammatory,
29 antibacterial, antioxidant, antiviral, anticancer, antipyretic, and analgesic activities, and has a
30 significant impact on the cardiovascular and cerebrovascular, digestive, respiratory, and central
31 nervous systems[1-4]. In the traditional production areas of *Hansenia*, the loss of germplasm
32 resources is an extremely serious problem. As the problem of artificial breeding has not been
33 solved, it is difficult to collect enough seeds, roots, and other reproductive materials in the
34 genuine production areas and even enough genetic standard materials, showing that the severity
35 of the risk of endangerment of *Hansenia* cannot be ignored. The wild-growing to domestic
36 cultivation for *Hansenia* and the establishment of a standardized production base are the most
37 effective ways to ensure the quality of medicinal materials, meet the medicinal requirements,
38 protect wild resources and achieve sustainable development. Due to the wide distribution range
39 and low altitude of *H. forbesii*, the requirements for environmental and soil conditions are
40 relatively loose, the root system is well developed and the medicinal value is the same as that of
41 *H. weberbaueriana*. Therefore, vigorously promoting the artificial cultivation of *H. forbesii* can
42 reduce the pressure on this resource.
43 The main distribution types of *H. forbesii* are purple stem type, green stem type, large leaf
44 type, small leaf type, root single branch type, root dispersed type, and other mixed species. The
45 excellent varieties of *H. forbesii* have not been popularised and applied in production and
46 cultivation, which cannot meet the needs of its industrial development. Moreover, there is a
47 serious problem of mixed varieties in field production, which has a significant impact on its
48 high-yield cultivation and is the bottleneck for the development of the *H. forbesii* industry.
49 Before this study, we tried to address these issues by the following actions.
50 Taking the wild *H. forbesii* (Wei-Qiang) resources in Gansu Province as the original materials,
51 using the single plant optimization method and taking the plant agronomic characters,
52 susceptibility, and drug grade as the indicators, we have selected two new varieties of *H. forbesii*
53 (Long-Qiang and Qiang-Xuan) with high quality and high yield through line identification, line
54 comparison, multi-point test, and production test. It is not clear what kind of variation exists at
55 the gene level and what is their phylogenetic relationship.
56 Chloroplast is an essential organelle in plant cells and it plays an important role in
57 photosynthesis, carbon sequestration, and amino acid synthesis. Its complete genome contains a
58 large amount of genetic information and its structure is highly conserved. It is widely used in the
59 study of plant molecular evolution and phylogeny, as well as in genetic transformation, genetic
60 engineering, and molecular breeding[5-7]. Recently, with the gradual popularization and low cost
61 of high-throughput sequencing technology, some scientists have proposed that chloroplast (cp)
62 genome	data	can	be	used	for	species	identification	and	interspecific	differentiation
63 research[8-12].
64 To find the gene hypervariable regions of three varieties of *H. forbesii* and determine their
65 phylogenetic relationship, we used Illumina hiseq technology to sequence the cp genomes. And
66 we compared the characteristics of cp genome, screened the different nucleotide sequences, and
67 deciphered the genetic relationship to provide the basis for the differentiation of target varieties
68 with excellent and optimal shape.

# 69 2. Materials and methods

## 70 2.1. Materials and pretreatment

71 The fresh, tender and healthy leaves of three *H. forbesii* were collected from the germplasm
72 resource garden in Taoyang Town, Lintao County, Gansu Province in China on 30 September
73 2021. Professor Zhirong Sun, a scientist in the national traditional Chinese medicine system,
74 identified that all the samples were *Hansenia forbesii* H.Boissieu (Apiaceae). The aboveground
75 forms of three *H. forbesii*, are shown in Fig. 1 and described in Table 1, which named QX, LQ,
76 and WQ according to their morphological characteristics and breeding process. After cutting off
77 the veins of leaves with clean scissors, we put the remaining leaves into molecular bags, added
78 silica gel, and saved. The voucher specimens (QH210930) of all materials were kept in the herbal
79 medicine library of the school of Chinese Materia Medica, Beijing University of Chinese
80 Medicine.
81 **Table 1 The aboveground forms of three *H. forbesii.***

|  | | Leaves |  |  | Stems |  |  |  | Raceme |  | Branches |
| --- | --- | --- | --- | --- | --- | --- | --- | --- | --- | --- | --- |
| Name | Length  (mm) | Width  (mm) | Strigose | Colour | Length  (cm) | Strigose | Colour | Length  (mm) | Width  (mm) | Flower | Amount (piece) |
| LQ | 61-93 | 32-51 | None | Purple | 78-121 | None | Purple | 78-101 | 107-251 | Umbrella-type | 21-30 |
| QX | 67-89 | 32-54 | None | Green | 81-125 | None | Yellow-green | 90-110 | 113-250 Umbrella-type | | 21-30 |
| WQ | 67-90 | 29-54 | None | Green | 77-129 | None | Yellow-green | 89-111 | 114-254 Umbrella-type | | 20-29 |

## 82 2.2. Total DNA extraction and Library Preparation

83 The total DNA was extracted using a small plant DNA Extraction Kit (Omega Bio-Tek, USA).
84 Then we randomly broke qualified DNA samples using an ultrasonic crusher (Covaris, USA),
85 and then completed the whole library preparation through the steps of end repair, adding
86 A-tailing, adding sequencing connector, purification, and PCR amplification. The qualified
87 library was sequenced using the Illumina high-throughput sequencing platform (Illumina,
88 NovaSeq 6000, USA).

## 89 2.3. Chloroplast genome assembly and annotation

90 The high-quality reads were assembled using Get Organelle v.4.0 and then annotated by
91 CpGAVAS2[13]. The annotations of tRNA genes were confirmed by using tRNAscan-SE

92	v.2.03[14].

93 The cp genomes of LQ, QX, and WQ were submitted to GenBank at the National Center of
94 Biotechnology Information (NCBI), and the accession numbers were ON 208134, ON208135,
95 and ON208136, respectively. Fully annotated plastome circular diagrams were drawn by
96 OrganellarGenomeDRAW (OGDRAW)[15].

## 97 2.4. Codon usage

98 The protein-coding genes were extracted by Phylosuite v.1.2.2[16]. Relative synonymous codon
99 usage (RSCU) and codon usage values were analyzed by CodonW v.1.4.2. Moreover, the RSCU
100 values were shown in a heatmap by TBtools[17].

## 101 2.5. Repeat analysis and comparative analyses

102 Repetitive sequence analyses were performed using CPGAVAS2 analysis. Tandem repeats
103 were identified using default settings by Tandem Repeats Finder[18]. The Misa.pl was used to
104 screen the simple sequence repeats (SSRs)[19]. The scattered repetitive sequences were found by
105 using VMATCH. The REPuter was used to determine the size and location of the oligonucleotide
106 repeats (ORs)[20]. The complete cp genomes of three cultivated varieties were compared by
107 mVISTA[21], and the genome of *H. forbesii* (NC035054) was used as the reference sequence for
108 annotation. Sliding window analysis was conducted to assess the nucleotide diversity (Pi) values
109 of the cp genomes by DnaSP v6 (window length = 300 bp, step size = 25 bp). IRscope[22] was
110 used to analyze inverted repeated traction and expansion at cp genomes’ junctions.

## 111 2.6. Phylogenetic analysis

112 Phylogenetic analysis was performed based on 37 complete cp genomes, including the three
113 assembled sequences in our study, 34 cp genomes (30 Genus, 2 Family) downloaded from the
114 NCBI and *Cornus officinalis* (NC042746) as outgroup. The shared protein-coding genes were
115 extracted using Phylosuite v.1.2.2 software[16], and the MAFFT v.7.307 was used for
116 alignment[23]. Subsequently, the alignment was conducted using the GTR+I+Gevolution model
117 based on Bayesian inference (BI) in MrBayes [24]. The parameter was set to run for five million
118 generations and sampled every 1,000 generations, with all other settings left at their defaults, and
119 the first 25% of each run was discarded as burn-in. The alignment was also evaluated using
120 bootstrap analysis on 1000 in a maximum likelihood (ML) by RAxML (Stamatakis, 2014), with
121 parameters: raxmlHPC-PTHREADS-SSE3 -fa -N 1000-m GTRGAMMA -x 551,314,260 -p
122 551,314,260 -o *Cornus*_*officinalis* _NC_042746-T 20, 1000 replications and best-fit model
123 selection.

# 124 3. Results

## 125 3.1. Chloroplast genome information

126 The size and GC content of these genomes have been analyzed (Table 2). The length of the cp
127 genome of *H. forbesii* QX was the longest in three varieties (157,181 bp), the total GC content of
128 three cp genomes was about 37.6%, and they all had a typical four-region structure, in which the
129 LSC (large single-copy region) was 86,167 bp (LQ), 86,361 bp (QX), and 86,188 bp (WQ), the
130 SSC (small single-copy region) was 17,755 bp (LQ), 17,786 bp (QX) and 17,755 bp (WQ) and
131 the IR (inverted repeats, including IRa and IRb) was 26,516 bp (LQ), 26,517 bp (QX), and
132 26,516 bp (WQ) (Fig 2). A total of 129 genes were found in these varieties, including 84
133 protein-coding genes, 37 tRNA genes, and 8 rRNA genes (Table 3). The number and types of
134 introns were similar among the three *H. forbesii*. The four types of cp genes contained 45
135 photosynthesis-related genes, 25 self-replication-related genes, six other genes, and three
136 unknown genes. Amongst them, *ndh*B, *rpl*2, *rpl*23, *rps*12, *rps*7*,* and *ycf*2 appeared twice. Eleven
137 genes each contained one intron, including *rpl*2 (×2) and *ndh*B (×2), which were located in the
138 IR, and the genes (*rps*16, *atp*F, *rpo*C1, *pet*B, *pet*D, and *rpl*16) were located in the LSC, and the
139 *ndh*A was the only present in the SSC region. In addition, the *ycf*3 and *clp*P comprise two
140 introns.

## 141 Table 2 Basic information of cp genomes of three varieties of *H. forbesii*.

| Name | Overall length | LSC | SSC | IR | GC  content (%) | protein-coding genes | tRNA  genes | rRNA  genes |
| --- | --- | --- | --- | --- | --- | --- | --- | --- |
| *H.f.* LQ | 156,954 | 86,167 | 17,755 | 26,516 | 37.68 | 84 | 37 | 8 |
| *H.f.* QX | 157,181 | 86,361 | 17,786 | 26,517 | 37.66 | 84 | 37 | 8 |
| *H.f.* WQ | 156,975 | 86,188 | 17,755 | 26,516 | 37.68 | 84 | 37 | 8 |

142 GC: Percentage of G/C base number in total base number.

## 143 Table 3 List of genes in the cp genome of three varieties of *H. forbesii*.

Category of genes	Group of genes	Name of genes	Number

*rrn*16S (×2), *rrn*23S (×2), *rrn*5S (×2),

rRNA	rRNA

8

*rrn*4.5S (×2)

tRNA	tRNA

*trn*Y-GUA, *trn*W-CCA, **trn*V-UAC,

*trn*V-GAC(×2), *trn*T-UGU,

*trn*T-GGU(×2), *trn*S-UGA, *trn*S-GGA,

*trn*S-GCU, **trn*S-CGA, *trn*R-UCU, *trn*R-ACG(×2), *trn*Q-UUG, *trn*P-UGG,

37

*trn*N-GUU(×2), *trn*M-CAU(×4),

*trn*L-UAG,* *trn*L-UAA, *trn*L-CAA(×2),* *trn*K-UUU, *trn*H-GUG, *trn*G-GCC,

*trn*F-GAA, **trn*E-UUC(×3), *trn*D-GUC, *trn*C-GCA,**trn*A-UGC(×2)

Subunits of ATP synthase	*atp*A, *atp*B, *atp*E, **atp*F, *atp*H, *atp*I	6

*psb*A, *psb*B, *psb*C, *psb*D, *psb*E, *psb*F, *psb*I,

Subunits of photosystem II

*psb*J, *psb*K, *psb*L, *psb*M, *psb*N, *psb*T,	15

*psb*Z, ***ycf*3

**ndh*A, **ndh*B (×2), *ndh*C, *ndh*D, *ndh*E,

Genes for photosynthesis

Subunits of NADH-dehydrogenase

12

*ndh*F, *ndh*G, *ndh*H, *ndh*I, *ndh*J, *ndh*K

Subunits of cytochrome b/f complex	*pet*A, **pet*B, **pet*D, *pet*G, *pet*L, *pet*N	6

Subunits of photosystem I	*psa*A, *psa*B, *psa*C, *psa*I, *psa*J	5

Subunit of rubisco	*rbc*L	1

*rpl*14, **rpl*16, **rpl*2(×2), *rpl*20, *rpl*22,

Large subunit of ribosome

11

*rpl*23(×2), *rpl*32, *rpl*33, *rpl*36

Self replication

DNA dependent RNA polymerase	*rpo*A, *rpo*B, **rpo*C1, *rpo*C2	4

*rps*11, *rps*12(×2), *rps*14, *rps*15, **rps*16,

Small subunit of ribosome

*rps*18, *rps*19, *rps*2, *rps*3, *rps*4, *rps*7(×2),	14

*rps*8

Subunit of Acetyl-CoA-carboxylase	*acc*D	1

c-type cytochrom synthesis gene	*ccs*A	1

Other genes

Envelop membrane protein	*cem*A	1

Protease	***clp*P	1

Translational initiation factor	*inf*A	1

Maturase	*mat*K	1

Unkown	Conserved open reading frames	*ycf*1, *ycf*2 (×2), *ycf*4	4

144 *Gene with one intron, **Gene with two introns.
145 145

## 146 3.2. Comparison of chloroplast introns and exons

147 By analyzing the cp genome annotation files of the three varieties of *H. forbesii*, we found that
148 the introns and exons of 21 genes in the cp genomes of the three varieties were similar. Amongst
149 them, in the *trn*K*-*UUU, *rps*16, *atp*F*, ycf*3, *clp*P*,* and *rpl*16, QX had one more bp than LQ and
150 WQ, but one less bp in *rpo*C1 gene (S Table 1). Studies have shown that introns can reduce gene
151 splicing and translation, slow down cell metabolism and reduce energy consumption and the Clp
152 protease gene is also related to plant disease resistance [25], which may be the reason why QX
153 has better disease resistance than LQ and WQ.

## 154 3.3. Codon usage

155 The cp genome of three *H. forbesii* contained 64 codons encoding 20 amino acids. The result
156 of the RSCU revealed that 32 codons were used frequently in these cultivars, with the highest
157 frequency of UUA followed by AGA (Fig 3). Moreover, the codon exhibited a strong bias toward
158 an A or T at the third position. The codons that contain A/T at the 3’ end mostly have RSCU ≥1,
159 whereas the codons are having C or G at the 3’ end mostly have RSCU ≤1. Amino acid
160 frequency analyses revealed the highest frequency of Leucine, whereas Tryptophane was a rare
161 amino acid. In general, we found high similarities in codon usage and amino acid frequency
162 among the three cultivated varieties, and both contain high AT content.

## 163 3.4. Repeat analysis

164 Our analyses identified SSRs per genome composed of mono- to di- nucleotide repeating units
165 (Fig 4A). The number and type of SSRs in LQ and WQ were similar, with 34 single nucleotide
166 repeats and 2 dinucleotide repeats. QX contains 39 single nucleotide repeats more than LQ and
167 WQ. Moreover, the main type of mononucleotide repeats was T. Oligonucleotide repeats in three
168 varieties. Fig 4B showed that the number of repeats varied in three varieties. We discovered 49
169 repeats in three varieties (LQ: 26 forward and 23 palindromic, QX: 30 forward and 19
170 palindromic, and WQ: 26 forward and 23 palindromic). Most of the repeats ranged in size from
171 30 to 40 bp. This result showed that QX were more different than LQ and WQ. We also
172 evaluated the number of repeats about the species' phylogenetic position using the topology in
173 Fig. 8.

## 174 3.5. Inverted repeats contraction and expansion

175 The inverted repeats contraction and expansion revealed variation at LSC/IRs/SSC junctions
176 (Fig 5). In three varieties, a truncated copy of the *rps*19 gene was found at the IRb/LSC junction;
177 the *rpl*22 gene was found entirely in the LSC region; and the *rpl*2 gene was found entirely in the
178 IRb region. Another truncated copy of *ndh*F gene was found at the junction of IRb/SSC in all
179 species, which starts in IRb regions and integrates into the SSC region. Moreover, a truncated
180 copy of *ycf*1 was found in SSC/IRa junction, which was longer in IRa of *H. forbesii*. In three
181 varieties, *trn*H completely exists in LSC and only 3 bp from the junction of IRa/LSC. These
182 results showed that the chloroplast genome of these three varieties did not expand or contract.

## 183 3.6. Comparative cp genomic analysis

184 The cp genomes of the three varieties were compared by mVISTA, and the *H. forbesii*
185 (NC035054) was used as the reference sequence for annotation. The three varieties exhibited
186 similar variation sites and degrees of variation (Fig 6). The coding regions (CDS) were more
187 conserved than the intergenic spacers (IGS). The high divergence area in IGS was found in
188 *pet*N-*psb*M, *trn*Y(GUA)-*trn*T(GGU), *ndh*C-*trn*V(UAC), *pet*A-*psb*J, *rps*8-*rpl*14, *rrn*4.5-*rrn*5.
189 Furthermore, some mutations of CDS were found in *rpo*C1 and *rpl*2. Moreover, the result
190 showed that IR regions had lower sequence divergence than LSC and SSC regions.
191 We used the DnaSP software to compare the nucleotide variation values (Pi) between the
192 whole cp genomes of the three varieties. The hypervariable regions were detected, and the
193 sequence differences were analyzed. Sliding window analysis revealed that the nucleotide
194 diversity values varied from 0 to 0.008. Three mutational hotspots in the LSC and SSC regions
195 were identified, including *atp*F-*atp*H, *pet*D, and *rps*15-*ycf*1 (Fig 7). These can be used as
196 potential sites for studying population genetics and the identification of these three varieties.

## 197 3.7. Phylogenetic analysis

198 The phylogenetic trees (ML and BI) were constructed by the shared protein-coding genes to
199 investigate the phylogeny of the three *H. forbesii*, and *C. officinalis* (NC042746) was the
200 outgroup. Most nodes were supported with maximum support (100% bootstrap support) on the
201 consensus trees, and the topological structure of the two phylogenetic trees had perfect
202 consistency (Fig 8). Three varieties of *H. forbesii* clustered with *Hansenia forbesii* (NC035054)
203 and then merged with *H. forrestii* (NC035056) and *H. oviformis* (NC035055), indicating that LQ
204 and WQ were closely related, followed by QX, and they were closely related to *Hansenia*
205 *forbesii* (NC035054). Finally, they became a clade with *Changium smyrnioides* (NC053938),
206 *Tongoloa silaifolia* (NC049062), *H. weberbaueriana* (NC035053), *Haplosphaera himalayensis*
207 (NC056096). This clade had a close genetic relationship, in which QX was relatively distant
208 from LQ and WQ.

# 209 4. Discussion and Conclusion

210 This study found that the three varieties of *Hansenia forbesii* had similar genetic relationships
211 and there were no different functional genes in the cp genome. Only the number of introns of
212 some *H. forbesii* QX genes was higher than *H. forbesii* LQ and *H. forbesii* WQ. We found three
213 hypervariable regions between cp genomes of the three varieties of *H. forbesii*, including
214 *atp*F-*atp*H, *pet*D, and *rps*15-*ycf*1. So the character differences between the three varieties of *H.*
215 *forbesii* could be judged by the complete cp genome. The IR regions exhibited lower sequence
216 divergence than LSC and SSC regions and were consistent with the previous comparisons of cp
217 genomes [8, 26]. Intergenic spacer regions are the most frequently used cp markers for
218 phylogenetic studies at lower taxonomic levels in plants [27], as they are regarded as more
219 variable and could provide more phylogenetically informative characters. In a previous study,
220 *rps*15-*ycf*1 was identified as a mutational hotspot for identifying *Isodon rubescens* [10]. The
221 *Pet*D located in the LSC region, as a protein-coding gene, contains an intron, which is consistent
222 with previous genetic studies [26] and can also be used as a highly variable region for species
223 identification. Phylogenetic analysis further showed that *H. forbesii* LQ and *H. forbesii* WQ were
224 closely related, followed by *H. forbesii* QX, and they were closely related to *Hansenia forbesii*
225 (NC035054), this also indirectly indicated the reliability of the data of three varieties of *H.*
226 *forbesii*.
227 Chen reported the cp genome sequences of six species of *Ligularia* with similar morphology
228 and found that there was no potential coding or non-coding region to distinguish them effectively,
229 but the whole cp genome could be used as a super barcode to identify these species [28]. Fu
230 obtained 48 cp genomes from 16 *Taxus* species and *Pseudotaxus chienii* and constructed the ML
231 evolutionary tree, based on the whole genome sequence, which showed that it can successfully
232 distinguish all *Taxus* species and can be used as a super barcode to identify species [29]. Plant
233 genomes include the nuclear genome, mitochondrial genome, and chloroplast genome.
234 Compared with the mitochondrial genome and nuclear genome, the cp genome has a unilineal
235 inheritance and its size, structure, and gene species are relatively conservative[30-33]. We found
236 that the internal reason for the difference in the external phenotypes of the three varieties of *H.*
237 *forbesii* can be reflected in the highly variable region of the cp genome, so the complete cp
238 genomes of three varieties of *H. forbesii* can be used as the DNA super barcode for identifying
239 these three varieties. In addition, the genetic relationship of the *H. forbesii* QX variety is far
240 away from the other two varieties, so it can be selected as the first generation variety for single
241 plant optimization and pure breeding to ensure its excellent phenotypes, and it can be
242 popularized and applied on a large area. This study can provide a reference for the sustainable
243 utilization of *Hansenia forbesii* resources and the identification of different varieties with
244 different phenotypes of the same species.

# 245 Declaration of Competing Interest

246 We declare that there is no conflict of interest regarding the publication of this article.

# 247 Acknowledgments

248 This research was supported by the Ministry of Finance and Ministry of Agriculture and Rural
249 Areas: special subsidy of national modern agricultural industrial technology system (cars-21);
250 Gansu Dingxi Science and Technology Project (dx2018n04).

# 251 Data availability statement

252 The genome sequence data that support the findings of this study are openly available in
253 GenBank of NCBI at [https://[www.ncbi.nlm.nih.gov]](http://www.ncbi.nlm.nih.gov/) under accession no. ON 208134,

254	ON208135, ON208136.

# 255 Author Contributions

256 **Data curation:** Chenghao Zhu.
257 **Formal analysis:** Chenghao Zhu, Yuan Jiang, Yu Bai.
258 **Funding acquisition:** Shengjian Dong, Zhirong Sun.
259 **Methodology:** Yuan Jiang.
260 **Resources:** Chenghao Zhu, Yu Bai.
261 **Software:** Yuan Jiang.
262 **Writing – original draft:** Chenghao Zhu.
263 **Writing – review & editing:** Yuan Jiang, Yu Bai, Shengjian Dong, Zhirong Sun.

# 264 References

265 1.	Li YH, Jiang SY, Guan YL, Liu X, Zhou Y, Li LM, et al. Quantitative determination of the
266 chemical profile of the plant material "Qiang-huo" by LC-ESI-MS-MS. Chromatographia.

267	2006;64(7-8):405-11. https://doi.org/10.1365/s10337-006-0052-2.

268 2.	Shi J, Jiang SY, Ma XJ, Sun H, Zhou Y. Studies on seeds germination and seedlings growth
269 of Notopterygium incisum. Zhongguo Zhong Yao Za Zhi. 2007;32(18):1841-4. PMID:

270	18051884.

271 3.	Xu KJ, Jiang SY, Zhou Y, Zhang YX, Xia B, Xu XM, et al. Discrimination of the seeds of
272 Notopterygium incisum and Notopterygium franchetii by validated HPLC-DAD-ESI-MS
273 method and principal component analysis. J Pharmaceut Biomed. 2011;56(5):1089-93.

274	[https://doi.org/10.1016/j.jpba.2011.07.034.](https://doi.org/10.1016/j.jpba.2011.07.034) PMID: 21856104..

275 4. Azietaku JT, Ma HF, Yu XA, Li J, Oppong MB, Cao J, et al. A review of the
276 ethnopharmacology, phytochemistry and pharmacology of Notopterygium incisum. J 277	Ethnopharmacol. 2017;202:241-55. [https://doi.org/10.1016/j.jep.2017.03.022.](https://doi.org/10.1016/j.jep.2017.03.022) PMID: 278	28336469.
279 5. Jin Y, Zhang T, Liu B, Zheng C, Huo H, Zhang J. Comparative and phylogenetic analysis of
280 the complete	chloroplast	genome	sequences	of	Allium	mongolicum. Sci	Rep.

281	2022;12(1):21676. https://doi.org/10.1038/s41598-022-26354-0. PMID: 36522492.

282 6. Fahrenkrog AM, Matsumoto GO, Toth K, Jokipii-Lukkari S, Salo HM, Haggman H, et al.
283 Chloroplast genome assemblies and comparative analyses of commercially important
284 Vaccinium	berry	crops.	Sci	Rep.	2022;12(1):21600.

285	https://doi.org/10.1038/s41598-022-25434-5. PMID: 36517490.

286 7. Zhang Q, Belyakov EA, Lapirov AG, Zhao Y, Freeland J, Xu X. A reappraisal of the
287 phylogeny and historical biogeography of Sparganium (Typhaceae) using complete
288 chloroplast	genomes.	BMC	Plant	Biol.	2022;22(1):588.

289	https://doi.org/10.1186/s12870-022-03981-3. PMID: 36522739.

290 8. Wang J, Qian J, Jiang Y, Chen XC, Zheng BJ, Chen SL, et al. Comparative Analysis of
291 Chloroplast Genome and New Insights Into Phylogenetic Relationships of Polygonatum and
292 Tribe Polygonateae. Front Plant Sci. 2022;13. [https://doi.org/10.3389/fpls.2022.882189.](https://doi.org/10.3389/fpls.2022.882189)

293	PMID: 35812916.

294 9. Kuang Q, Sheng WT. The entire chloroplast genome sequence of Asparagus setaceus
295 (Kunth) Jessop: Genome structure, gene composition, and phylogenetic analysis in 296	Asparagaceae. Open Life Sci. 2022;17(1):1541-54. [https://doi.org/10.1515/biol-2022-0497.](https://doi.org/10.1515/biol-2022-0497) 297	PMID: 36474705.
298 10. Zhou ZY, Wang J, Pu TT, Dong JJ, Guan Q, Qian J, et al. Comparative analysis of
299 medicinal plant Isodon rubescens and its common adulterants based on chloroplast genome
300 sequencing. Front Plant Sci. 2022;13. [https://doi.org/10.3389/fpls.2022.1036277.](https://doi.org/10.3389/fpls.2022.1036277) PMID:

301	36479509.

302 11. Zeng QW, Chen M, Wang SC, Xu XX, Li T, Xiang ZH, et al. Comparative and phylogenetic
303 analyses of the chloroplast genome reveal the taxonomy of the Morus genus. Front Plant Sci.

304	2022;13. [https://doi.org/10.3389/fpls.2022.1047592.](https://doi.org/10.3389/fpls.2022.1047592) PMID: 36507423.

305 12. Lan ZH, Shi YH, Yin QG, Gao RR, Liu CL, Wang WT, et al. Comparative and phylogenetic
306 analysis of complete chloroplast genomes from five Artemisia species. Front Plant Sci.

307	2022;13. [https://doi.org/10.3389/fpls.2022.1049209.](https://doi.org/10.3389/fpls.2022.1049209) PMID: 36479523.

308 13. Shi LC, Chen HM, Jiang M, Wang LQ, Wu X, Huang LF, et al. CPGAVAS2, an integrated
309 plastome sequence annotator and analyzer. Nucleic Acids Res. 2019;47(W1):W65-W73.

310	[https://doi.org/10.1093/nar/gkz345.](https://doi.org/10.1093/nar/gkz345) PMID: 31066451.

311 14. Chan PP, Lin BY, Mak AJ, Lowe TM. tRNAscan-SE 2.0: improved detection and functional
312 classification of	transfer	RNA	genes. Nucleic	Acids	Res. 2021;49(16):9077-96.

313	https://doi.org/10.1093/nar/gkab688. PMID: 34417604.

314 15. Greiner S, Lehwark P, Bock R. OrganellarGenomeDRAW (OGDRAW) version 1.3.1:
315 expanded toolkit for the graphical visualization of organellar genomes. Nucleic Acids Res.

316	2019;47(W1):W59-W64. https://doi.org/10.1093/nar/gkz238. PMID: 30949694.

317 16. Zhang D, Gao FL, Jakovlic I, Zou H, Zhang J, Li WX, et al. PhyloSuite: An integrated and
318 scalable desktop platform for streamlined molecular sequence data management and
319 evolutionary	phylogenetics	studies. Mol	Ecol	Resour. 2020;20(1):348-55.

320	https://doi.org/10.1111/1755-0998.13096. PMID: 31599058.

321 17. Chen CJ, Chen H, Zhang Y, Thomas HR, Frank MH, He YH, et al. TBtools: An Integrative
322 Toolkit Developed	for	Interactive	Analyses	of	Big	Biological	Data. Mol	Plant.

323	2020;13(8):1194-202. https://doi.org/10.1016/j.molp.2020.06.009. PMID: 32585190.

| 324 | 18. | Benson G. Tandem repeats finder: a program to analyze DNA sequences. Nucleic Acids Res. |
| --- | --- | --- |
| 325 |  | 1999;27(2):573-80. Epub 1998/12/24. https://doi.org/10.3390/plants11162103. PMID: |
| 326 |  | 9862982. |
| 327 | 19. | Beier S, Thiel T, Munch T, Scholz U, Mascher M. MISA-web: a web server for |
| 328 |  | microsatellite prediction. Bioinformatics. 2017;33(16):2583-5. |
| 329 |  | https://doi.org/10.1093/bioinformatics/btx198. PMID: 28398459. |
| 330 | 20. | Kurtz S, Choudhuri JV, Ohlebusch E, Schleiermacher C, Stoye J, Giegerich R. REPuter: the |
| 331 |  | manifold applications of repeat analysis on a genomic scale. Nucleic Acids Res. |
| 332 |  | 2001;29(22):4633-42. https://doi.org/10.1093/nar/29.22.4633. PMID: 11713313. |
| 333 | 21. | Frazer KA, Pachter L, Poliakov A, Rubin EM, Dubchak I. VISTA: computational tools for |
| 334 |  | comparative genomics. Nucleic Acids Res. 2004;32:W273-W9. |
| 335 |  | https://doi.org/10.1093/nar/gkh458. PMID: 15215394. |
| 336 | 22. | Amiryousefi A, Hyvonen J, Poczai P. IRscope: an online program to visualize the junction |
| 337 |  | sites of chloroplast genomes. Bioinformatics. 2018;34(17):3030-1. |
| 338 |  | https://doi.org/10.1093/bioinformatics/bty220. PMID: 29659705. |
| 339 | 23. | Katoh K, Standley DM. MAFFT Multiple Sequence Alignment Software Version 7: |
| 340 |  | Improvements in Performance and Usability. Mol Biol Evol. 2013;30(4):772-80. |
| 341 |  | [https://doi.org/10.1093/molbev/mst010.](https://doi.org/10.1093/molbev/mst010) PMID: 23329690. |
| 342 | 24. | Nylander JAA, Ronquist F, Huelsenbeck JP, Nieves-Aldrey JL. Bayesian phylogenetic |
| 343 |  | analysis of combined data. Syst Biol. 2004;53(1):47-67. |
| 344 |  | [https://doi.org/10.1080/10635150490264699.](https://doi.org/10.1080/10635150490264699) PMID: 14965900. |
| 345 | 25. | Parenteau J, Maignon L, Berthoumieux M, Catala M, Gagnon V, Abou Elela S. Introns are |
| 346 |  | mediators of cell response to starvation. Nature. 2019;565(7741):612-+. |
| 347 |  | https://doi.org/10.1038/s41586-018-0859-7. PMID: 30651641. |
| 348 | 26. | Jiang Y, Miao YJ, Qian J, Zheng Y, Xia CL, Yang QS, et al. Comparative analysis of |
| 349 |  | complete chloroplast genome sequences of five endangered species and new insights into |
| 350 |  | phylogenetic relationships of Paris. Gene. 2022;833. |
| 351 |  | https://doi.org/10.1016/j.gene.2022.146572. PMID: 35609799. |
| 352 | 27. | Shaw J, Lickey EB, Beck JT, Farmer SB, Liu WS, Miller J, et al. The tortoise and the hare |
| 353 |  | II: Relative utility of 21 noncoding chloroplast DNA sequences for phylogenetic analysis. |
| 354 |  | Am J Bot. 2005;92(1):142-66. https://doi.org/10.3732/ajb.92.1.142. PMID: 21652394. |
| 355 | 28. | Chen XL, Zhou JG, Cui YX, Wang Y, Duan BZ, Yao H. Identification of Ligularia Herbs |
| 356 |  | Using the Complete Chloroplast Genome as a Super-Barcode. Front Pharmacol. 2018;9. |
| 357 |  | https://doi.org/10.3389/fphar.2018.00695. PMID: 30034337. |

358 29. Fu CN, Wu CS, Ye LJ, Mo ZQ, Liu J, Chang YW, et al. Prevalence of isomeric plastomes
359 and effectiveness of plastome super-barcodes in yews (Taxus) worldwide. Sci Rep. 360	2019;9(1):2773. Epub 2019/02/28. https://doi.org/10.1038/s41598-019-39161-x. PMID: 361	30808961.
362 30. Huang SA, Kang ZJ, Chen ZF, Deng YF. Comparative Analysis of the Chloroplast Genome
363 of Cardamine hupingshanensis and Phylogenetic Study of Cardamine. Genes-Basel.

364	2022;13(11). https://doi.org/10.3390/genes13112116. PMID: 36421792.

365 31. Gao Y, Shen GH, Yuan GH, Tian ZH. Comparative Analysis of Whole Chloroplast
366 Genomes of Three Common Species of Echinochloa (Gramineae) in Paddy Fields. Int J Mol

367	Sci. 2022;23(22). https://doi.org/10.3390/ijms232213864. PMID: 36430336.

368 32. Dorjee T, Gao F, Zhou YJ. The complete chloroplast genome of Thermopsis lanceolata:
369 genome structure and its phylogenetic	relationships	within	the	family	Fabaceae. 370	Mitochondrial	DNA	B.	2022;7(12):2076-80. 371	https://doi.org/10.1080/23802359.2022.2154623. PMID: 36530460.
372 33. Parmar R, Cattonaro F, Phillips C, Vassiliev S, Morgante M, Rajora OP. Assembly and
373 Annotation of Red Spruce (Picea rubens) Chloroplast Genome, Identification of Simple
374 Sequence Repeats, and Phylogenetic Analysis in Picea. Int J Mol Sci. 2022;23(23).

375	https://doi.org/10.3390/ijms232315243. PMID: 36499570.

376

377

378

## 379 Captions

380 **Fig. 1. The aboveground forms of three *H. forbesii.***
381 **Fig. 2. Cp genome map of three varieties of *H. forbesii*.** Genes lying outside the circle are
382 transcribed in the counterclockwise direction, while those insides are transcribed in the
383 clockwise direction. The coloured bars indicate different functional groups. The darker grey area
384 in the inner circle denotes GC content, while the lighter grey corresponds to the AT content of
385 the genome.
386 **Fig. 3. The RSCU values of all protein-coding genes for three varieties.** Colour key: the red
387 values indicate higher RSCU values, and the green values indicate lower RSCU values.
388 **Fig 4. Comparison of repeats in three varieties.** (a). SSR distributed situation in the cp
389 genomes of five species. (b). Long repeats classification of five species. P-palindromic repeats;
390 F- forward repeats.

## 391 Fig 5. Comparison of quadripartite junction sites in three varieties cp genomes. Gene

392 transcribed clockwise are presented below the track, whereas transcribed counterclockwise are
393 presented on top of the track. The start and end of each gene from the junctions have been shown
394 with arrows. The T scale bar above or below the track shows genes integrated from one region of
395 the cp to another. JLB (IRb /LSC), JSA (SSC/IRa), JSB (IRb/SSC), and JLA (IRa/LSC) denote
396 the junction sites between the quadripartite regions of the genome.

## 397 Fig 6. Comparison of three varieties of cp genomes using *H. forbesii* (NC035054) annotation

398 **as a reference.** The vertical scale indicates the percentage of identity, ranging from 50 to 100%.
399 The horizontal axis shows the coordinates within the cp genome. Genome regions are
400 colour-coded as exons, introns, and intergenic spacer (IGS), and the Gray arrows indicate the
401 direction of transcription of each gene. Annotated genes are displayed along the top.

## 402 Fig 7. Sliding window test of nucleotide diversity (Pi) in the multiple alignments of the three

403 ***H. forbesii* cp genomes**. Peak regions with a Pi value of>0.004 were labelled with loci tags of
404 the genic or intergenic region names.

## 405 Fig 8. BI and ML phylogenetic tree reconstruction containing the cp genomes of 37 plants.

406 The number on the branch indicates that the support rate of the ML tree is greater than 50%; The
407 *C. officinalis* were set as the outgroup.
408 **Table 1 The aboveground forms of three *H. forbesii.***

## 409 Table 2 Basic information of cp genomes of three varieties of *H. forbesii*.

410 **Table 3 List of genes in the cp genome of three varieties of *H. forbesii*.**

Figure	[Click here to access/download;Figure;Figure 1.tif](https://www.editorialmanager.com/pone/download.aspx?id=32685522&guid=c695ce8d-e631-42ed-ac54-cf529eef2f28&scheme=1)

Figure	[Click here to access/download;Figure;Figure 2.tif](https://www.editorialmanager.com/pone/download.aspx?id=32685525&guid=2ffbd5ba-601b-4390-96e5-c9d5d25b5592&scheme=1)

Figure	[Click here to access/download;Figure;Figure 3.tif](https://www.editorialmanager.com/pone/download.aspx?id=32685526&guid=1f6144d5-a64e-4363-adf0-5957b79f7a95&scheme=1)

Figure	[Click here to access/download;Figure;Figure 4.tif](https://www.editorialmanager.com/pone/download.aspx?id=32685531&guid=75c75675-7f1c-4388-9854-790ad9c74fb1&scheme=1)

Figure	[Click here to access/download;Figure;Figure 5.tif](https://www.editorialmanager.com/pone/download.aspx?id=32685528&guid=ca189ab2-3e8a-410d-9a99-76b43df7c4a5&scheme=1)

Figure	[Click here to access/download;Figure;Figure 6.tif](https://www.editorialmanager.com/pone/download.aspx?id=32685532&guid=c81391f4-274f-4122-917e-202abf58a600&scheme=1)

Figure	[Click here to access/download;Figure;Figure 7.tif](https://www.editorialmanager.com/pone/download.aspx?id=32685534&guid=44436a23-85f5-455f-aa3a-9f11b9cdef70&scheme=1)

Figure	[Click here to access/download;Figure;Figure 8.tif](https://www.editorialmanager.com/pone/download.aspx?id=32685535&guid=8648f3d5-af6f-43aa-8fe8-b96f3376bd16&scheme=1)

Supporting Information

[Click here to access/download](https://www.editorialmanager.com/pone/download.aspx?id=32685550&guid=e3fec57b-e084-46ab-b0b7-67e1702f8ca6&scheme=1)

[**Supporting Information** S table 1.xlsx](https://www.editorialmanager.com/pone/download.aspx?id=32685550&guid=e3fec57b-e084-46ab-b0b7-67e1702f8ca6&scheme=1)
